# A Proteomic Approach Suggests Unbalanced Proteasome Functioning Induced by the Growth-Promoting Bacterium *Kosakonia radicincitans* in Arabidopsis

**DOI:** 10.3389/fpls.2017.00661

**Published:** 2017-04-26

**Authors:** Katja Witzel, Suayib Üstün, Monika Schreiner, Rita Grosch, Frederik Börnke, Silke Ruppel

**Affiliations:** Leibniz Institute of Vegetable and Ornamental CropsGroßbeeren, Germany

**Keywords:** plant proteasome, plant growth-promoting bacteria, protein mass spectrometry, *rpn12a*, two-dimensional gel electrophoresis

## Abstract

Endophytic plant growth-promoting bacteria have significant impact on the plant physiology and understanding this interaction at the molecular level is of particular interest to support crop productivity and sustainable production systems. We used a proteomics approach to investigate the molecular mechanisms underlying plant growth promotion in the interaction of *Kosakonia radicincitans* DSM 16656 with *Arabidopsis thaliana*. Four weeks after the inoculation, the proteome of roots from inoculated and control plants was compared using two-dimensional gel electrophoresis and differentially abundant protein spots were identified by liquid chromatography tandem mass spectrometry. Twelve protein spots were responsive to the inoculation, with the majority of them being related to cellular stress reactions. The protein expression of 20S proteasome alpha-3 subunit was increased by the presence of *K. radicincitans*. Determination of proteasome activity and immuno blotting analysis for ubiquitinated proteins revealed that endophytic colonization interferes with ubiquitin-dependent protein degradation. Inoculation of *rpn12a*, defective in a 26S proteasome regulatory particle, enhanced the growth-promoting effect. This indicates that the plant proteasome, besides being a known target for plant pathogenic bacteria, is involved in the establishment of beneficial interactions of microorganisms with plants.

## Introduction

Endophytic bacteria colonize the rhizosphere, phyllosphere and reproductive organs of plants (Compant et al., 2010; Bodenhausen et al., 2013), with some of them being able to stimulate plant growth [plant growth-promoting bacteria (PGPB)] and increase the plants fitness (suppressive bacteria). In general, the physiological alterations within the plant and the emerging phytostimulation provoked by PGPB are assigned to bacterial nitrogen fixation, increased nutrient uptake, production of plant hormones and modulation of plant development (van Loon, 2007; De-la-Pena and Loyola-Vargas, 2014). The plant root system has a vital role in water and nutrient acquisition, as well as in signaling of environmental cues and adaptation to altered conditions in the rhizosphere. Induction of root growth by PGPB is frequently observed and the enhanced nutritional plant status is often associated with an increased root system. However, it is largely unclear how morphogenetic processes shaping root length and root surface area are influenced by microorganisms. The microbial production of phytohormones is frequently reported, especially auxin, but functional proof using knock-out mutant strains is still scarce (Patten and Glick, 2002; Sun et al., 2009). Recent studies used the model plant *Arabidopsis thaliana* for dissecting the molecular background of growth-promotion upon PGPB inoculation. The effect of bacterial colonization on global plant gene expression was analyzed for *Bacillus subtilis* FB17 (Lakshmanan et al., 2013), *Burkholderia phytofirmans* PsJN (Poupin et al., 2013), *Pseudomonas thivervalensis* MLG45 (Cartieaux et al., 2003), *Pseudomonas fluorescens* strains (Verhagen et al., 2004; Wang et al., 2005; van de Mortel et al., 2012; Weston et al., 2012) and *Pseudomonas* sp. G62 (Schwachtje et al., 2011). Despite the wealth of transcriptional data, only little attempt has been made to characterize the observed transcriptional changes on the proteome level to shed more light on the plants adaptation to endophytic colonization (Jayaraman et al., 2012). In order to elucidate gene function, the investigation of the gene product, the protein, is inevitable. The proteome does not only provide a complementary level to the transcriptome for studying the plethora of responses between plants and PGPB, proteins are also, together with metabolites, directly related to a phenotypical manifestation of a physiological response (Feussner and Polle, 2015). Enhancements in proteomic technology related to protein separation and detection as well as mass spectrometry-based protein identification have an increasing impact on the study of plant responses to biotic interactions (Mathesius, 2009; Cheng et al., 2010). Furthermore, post-translational modifications of proteins, such as phosphorylation or glycosylation, generate a great diversity, complexity and heterogeneity of polypeptides (Nørregaard Jensen, 2004). To date, more than 300 post-translational modifications are known that control protein activity, interactions, localisation and turn-over, and their analysis represents one of the main challenges in proteomics. Recent studies indicate that presence of PGPB alters components of plant primary and secondary metabolism, thereby promoting plant growth and increasing its resistance (Du et al., 2016; Kwon et al., 2016).

We described the gram negative strain *Kosakonia radicincitans* (DSM 16656), formerly named as *Pantoea agglomerans* (Ruppel et al., 1992) and *Enterobacter radicincitans* (Witzel et al., 2012), which was isolated from the phyllosphere of winter wheat under temperate conditions (Ruppel, 1988). Growth promotion of root and shoot, along with increased yield, was conferred by inoculation of numerous crop and model plant species under controlled conditions or in the field (Höflich and Ruppel, 1994; Berger et al., 2013, 2015). Inoculation of *A. thaliana* resulted in increased rosette diameter and seed production (Brock et al., 2013). Scanning electron microscopy revealed that *K. radicincitans* colonizes the root surface, intercellular spaces of the root cortex, xylem vessels, and intercellular spaces of the mesophyll of winter wheat (Remus et al., 2000). Some indications on the biochemical background of observed growth-promoting effect were gained using pure bacterial culture. Biological nitrogen fixation was demonstrated (Ruppel and Merbach, 1995), as well as the solubilisation of low soluble phosphorous (Schilling et al., 1998). A possible interactive role with plant phytohormone status implies the bacterial production of auxins (indole-3-acetic acid, indole-3-lactic acid) and cytokinins (Scholz-Seidel and Ruppel, 1992). While the characterization of isolated PGPB using *in vitro* assays provided valuable insights into potential mechanisms underlying plant growth promotion, the mode of action *in planta* remains largely unknown. Also, little attention has been paid to proteome alterations in the host plant in response to colonization by PGPB. Thus, the present study aims at dissecting the consequence of *K. radicincitans* colonization on the protein complement of *A. thaliana* roots. The choice of the host plant was governed by recognition of available mutant libraries for *A. thaliana* that allow for functional characterization of putative candidates. Our intention was to identify differentially translated gene products by comparative proteome analysis carried out by two-dimensional (2D) gel electrophoresis. Emerging candidates from this analysis were more deeply investigated. Our data show that the host proteasome is affected by endophytic *K. radicincitans* colonization. Protein degradation is a fundamental biological process and in plants, proteolysis of misfolded, damaged and ubiquitin-labeled proteins is governed by the 20S and 26S proteasome. The 20S proteasome represents the catalytic core particle with proteolytic activity and together with the 19S regulatory particle, the 26S proteasome is formed (Kurepa and Smalle, 2008). The 19S regulatory particle controls ubiquitin-dependent protein degradation, while the free 20S proteasome removes oxidized proteins generated by the presence of reactive oxygen species. We used *A. thaliana* mutants deficient in 26S proteasome regulatory particles to unravel the effect of *K. radicincitans* on ubiquitin-dependent proteolysis.

## Materials and Methods

### Bacterial and Plant Growth Conditions

*Kosakonia radicincitans* DSM 16656 was cultivated overnight in standard nutrient broth (Merck, Germany) (Ruppel et al., 2006). The cells in the medium were pelleted by centrifugation. Cells were then washed twice by centrifugation with autoclaved 50 mM NaCl solution to remove medium components. Bacterial cells were diluted with physiological buffer solution (sterile 50 mM NaCl) to OD_620_ 0.2, which corresponds to a concentration of 10^9^cfu mL^-1^ and further diluted to give 10^7^ cfu mL^-1^. A 10 mL aliquot or 10 mL 50 mM NaCl was poured over the surface of each pot, and the plants were cultivated for a further 4 weeks.

*Arabidopsis thaliana* Oy-0, Col-0, *rpt2a-2, rpt12a-1* were grown on non-sterile standardized plant growth substrate (Fruhstorfer Erde type P, Germany) with a pH of 6.0 in a climate chamber under short-day conditions (8 h light/16 h dark, 22°C, 40–60% humidity). After 2 weeks, single plants were transferred into sand filled pots and inoculated with *K. radicincitans* DSM 16656. As control treatment, 50 mM NaCl was applied. Plants were watered with nutrient solution as described by Gibeaut et al. (1997) and after four more weeks, root tissue was harvested in liquid nitrogen. Plant growth measurements were taken of the root length, and the fresh weight of root and rosette of 20 plants, grown under control conditions or inoculated with *K. radicincitans*, in three independent experiments. Analysis for statistical significance was done using Student’s *t*-test implemented in SigmaPlot 12.3 software (SPSS, Inc., USA).

### Two-dimensional Gel Electrophoresis and Protein Identification

#### Protein Extraction for 2-D Gel Electrophoresis

Proteins were extracted from one g of homogenized frozen root material using phenolic extraction method (Faurobert et al., 2007). Briefly, proteins were extracted using a buffer containing 500 mM Tris-HCl, 50 mM EDTA, 700 mM sucrose, 100 mM KCl, 2% (v/v) β-mercaptoethanol, proteinase inhibitor. One volume of TE-buffered phenol was added and phase separation was achieved by centrifugation step (10 min, 5,500 *g*, 4°C). The phenolic phase was mixed again with 1 volume of extraction buffer. After centrifugation, proteins were precipitated from the phenolic phase using 0.1 M sodium acetate in methanol over night at -20°C. The solution was centrifuged as described above, the resultin protein pellets washed with 80% acetone and dried in a vacuum centrifuge. Protein pellets were dissolved in 8 M urea, 2% CHAPS, 20 mM DTT, 0.5% SERVALYT^TM^ Carrier Ampholytes pH 4–7 (SERVA Electrophoresis GmbH, Germany), as described in Witzel et al. (2009). The protein concentration was determined using the BradfordRED Kit (Expedeon, UK), which is compatible with the 2D resolving buffer, according to the manufacturer’s instructions.

#### 2-D GE and Protein Staining

Protein extracts were subjected to isoelectric focusing (IEF) and subsequent SDS-PAGE as described in Schlesier and Mock (2006). A 300 μg sample was loaded by rehydration onto immobilized pH gradient strip of 17 cm in length with a pH gradient of 4–7 (IPG BlueStrip, SERVA Electrophoresis GmbH, Germany). The separation on an PROTEAN^®^ i12^TM^ IEF System (Bio-Rad, USA) was performed with the following parameters: 15 h rehydration, 30 min gradient to 250 V, 2 h gradient to 10,000 V and hold at 10.000 V to a total of 50 kVh. Strips were equilibrated and electrophoresed according to Witzel et al. (2009). Briefly, after IEF, strips were equilibrated in buffer A (50 mM Tris/HCl, pH 8.8, 6 M urea, 30% v/v glycerin, 2% w/v SDS, 20 mM DTT, 0.01% bromphenol blue) and additionally in buffer B (50 mM Tris/HCl, pH 8.8, 6 M urea, 30% v/v glycerin, 2% w/v SDS, 135 mM iodoacetamide, 0.01% bromphenol blue) for 15 min each. The strips were then placed on top of an 11.25% SDS polyacrylamide gel and covered with 0.5% agarose. Separation in the second dimension was done by SDS-Page and afterward, gels were washed in water for 5 min and stained with colloidal Coomassie Brilliant Blue (InstantBlue, Expedeon, UK) following the manufacturer’s instructions. Three independent separations of each sample were performed to ensure technical reproducibility.

#### Image Analysis and Statistical Analysis of Two-dimensional Gel Patterns

Gel images were captured by a Perfection V700 Photo scanner (Seiko Epson Corporation, Japan). SameSpots v4.5 (TotalLab, UK) was used for image analysis. Gel images were automatically aligned and manually checked. Subsequent analysis of the aligned image set used algorithms implemented in the software to enable spot detection, background subtraction, normalization, and spot matching across experiments. One-way analysis of variance (ANOVA), implemented in the software, was used for differential expression analysis (*p* < 0.05).

#### Spot Identification

Selected protein spots were manually excised from the 2D gel, digested with trypsin as described by Witzel et al. (2007) and subjected to mass spectrometry. Briefly, after a washing step of 5 min, spots were reduced with 10 mM DTT in 25 mM ammonium bicarbonate for 1 h at 55°C under shaking conditions. Afterward, the solution was replaced by 55 mM iodoacetamide in 25 mM ammonium bicarbonate and the spot was incubated for 45 min at room temperature under shaking conditions in the dark. The gel plug was washed with 25 mM ammonium bicarbonate for 10 min, with 10 mM ammonium bicarbonate/50% acetonitrile for 30 min and with 25 mM ammonium bicarbonate. After the final washing step with 10 mM ammonium bicarbonate/50% acetonitrile for 30 min the spot was dried and digested with trypsin (Promega, USA), following the manufacturer’s instructions. Protein identification using nanoLC-ESI-MS/MS was performed by Proteome Factory (Proteome Factory AG, Germany). The MS system consisted of an Agilent 1100 nanoLC system (Agilent, Germany), PicoTip electrospray emitter (New Objective, USA) and an Orbitrap XL (ThermoFisher, Germany). Protein spots were in-gel digested by trypsin (Promega, Germany) and applied to nanoLC-ESI-MS/MS. Peptides were trapped and desalted on the enrichment column (Zorbax SB C18, 0.3 mm × 5 mm, Agilent) for 5 min using 2.5% acetonitrile/0.5% formic acid as eluent, then peptides were separated on a Zorbax 300 SB C18, 75 μm × 150 mm column (Agilent) using an acetonitrile/0.1% formic acid gradient. MS/MS spectra were recorded data-dependently by the mass spectrometer according to manufacturer’s recommendations. Proteins were identified using MS/MS ion search of the Mascot search engine (Matrix Science, UK) and NCBI nr protein database (subset: Green Plants; National Center for Biotechnology Information, USA). Ion charge in search parameters for ions from ESI-MS/MS data acquisition were set to “1+, 2+, or 3+” according to the instrument’s and method’s common charge state distribution. A 5 ppm peptide, 0.6 Da fragment tolerance, two missed cleavages and variable oxidation (Met), deamidated (NQ), and carbamidomethyl (Cys) were used as the search parameters. The same protein spot was excised from the root protein profiles of control and inoculated plants and only matching protein identifications were accepted.

### Proteasome Activity Measurement

Determination of proteasome activity was performed as described in Üstün et al. (2013) spectrofluorometrically using the fluorogenic substrate suc-LLVY-NH-AMC (Sigma-Aldrich, Germany). The same root material, as analyzed by 2DE, was used for measurement. In short, proteins in 100 mg of ground frozen root were extracted in 50 mM HEPES-KOH, pH 7.2, 2 mM ATP, 2 mM DTT, 250 mM sucrose. The total protein was quantified by Bradford protein assay (Bio-Rad, USA) using bovine serum albumin as a standard. Twenty-five μg of protein was mixed with proteolysis buffer (100 mM HEPES-KOH, pH 7.8, 5 mM MgCl_2_, 10 mM KCl, 2 mM ATP) and the reaction was initiated by adding 0.2 mM suc-LLVY-AMC. Released amino-methyl-coumarin (AMC) was measured using a fluorescence spectrophotometer (FLX800, BioTek), with an excitation wavelength of 360 nm and an emission wavelength of 460 nm. Analysis for statistical significance was done using Student’s *t*-test implemented in SigmaPlot 12.3 software (SPSS, Inc., USA).

### Protein Gel Blot Analysis

For detection of protein ubiquitinylation, root proteins were extracted as described for proteasome activity measurements. Twenty μg of protein per sample were added to loading buffer and proteins were separated on SDS-PAGE and transferred to PVDF-membranes. The blots were probed with the Arabidopsis UBQ11 antibody (Agrisera, Sweden) in a dilution of 1:5,000. After probing with secondary antibody horseradish peroxidase-labeled Goat anti-Rabbit IgG (H+L) in a dilution of 1:10,000, immunodetection was carried out using Pierce ECL Western Blotting Substrate (Thermo Fisher Scientific). Chemiluminescence of blots was captured using Octoplus QPLEX Fluorescence Imager (NH DyeAGNOSTICS, Germany). Western Blot lane intensities were quantified using ImageJ software^1^. Analysis for statistical significance was done using Student’s *t*-test implemented in SigmaPlot 12.3 software (SPSS, Inc., USA).

## Results

### Beneficial Plant Growth Responses

**FIGURE 1:**
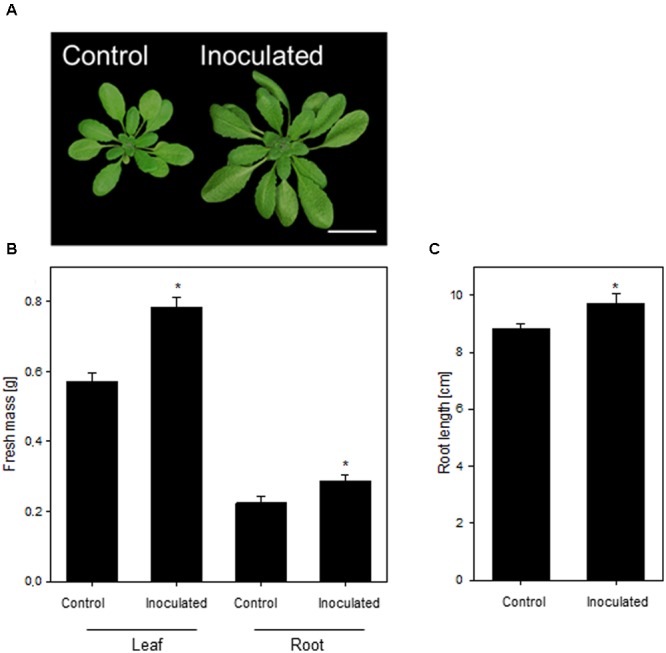
**The growth-promoting effect of *Kosakonia radicincitans* on *Arabidopsis thaliana***(A)**. (B)** The effect of colonization on leaf and root biomass. **(C)** Root length of differential treated plants. Values represent the mean ± SE (*n* = 20) and the asterisk indicate statistical differences between non-inoculated and inoculated plants (*p* < 0.05). Bar = 2 cm.

In this study, we aimed at dissecting the molecular adaptive processes during the stable establishment of *K. radicincitans* in *A. thaliana* Oy-0. This accession was isolated from a previous screen of *A. thaliana* genotypes and was found to be most responsive to the colonization of *K. radicincitans* (not shown). The growth-promoting activity of *K. radicincitans* on leaves of *A. thaliana* Col-0 has been shown earlier (Brock et al., 2013). In Oy-0, the leaf and root biomass was, respectively, 37 and 28% greater compared to non-inoculated plants 4 weeks after inoculation (**Figure 1B**). A beneficial effect of the inoculation was found also for root length, which increased by 10% (**Figure 1C**). **Figure 1** displays the outcome of one representative experiment out of three performed ones.

### Comparative Proteome Analysis

**FIGURE 2:**
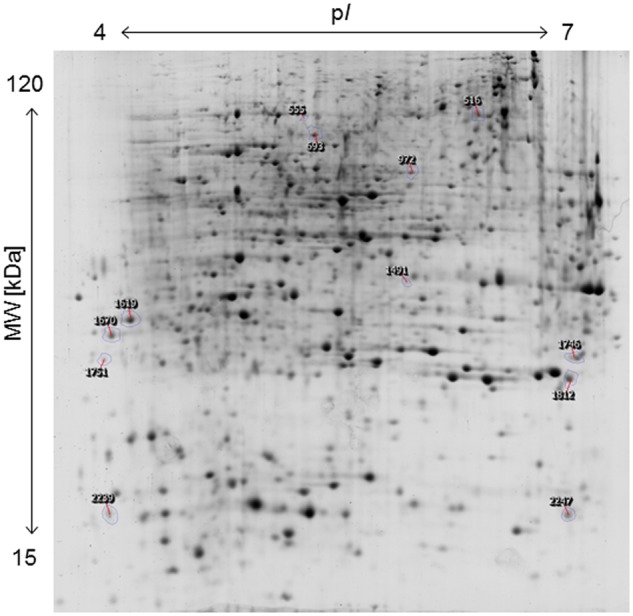
**Two-dimensional protein map of *A. thaliana* roots, indicating the position of spots identified by mass spectrometry.** Sample loading, protein separation, and visualization were as described in Section “Materials and Methods”.

The applied bacteria enter the host plant via roots and the endophytic establishment is likely to affect root physiology. Therefore, a proteome analysis of root tissue was conducted to characterize root proteins in response to endophytic colonization with *K. radicincitans*. Root samples from three independent experiments were separated on 2D gels and each sample was run in technical triplicates. Approximately, 1,700 protein spots were matched on 2D gels between biological and technical replicates. The comparative image analysis identified 12 differentially expressed spots between root samples of control and inoculated plants that were manually excised from 2D gels for tryptic digest and subsequent mass spectrometry-based identification (**Figure 2**). Identification was successful for all selected spots and was confirmed by analyzing spots from gels of control and inoculated root samples (**Table 1**). Detailed information on *de novo* sequencing data is provided in **Supplementary Table S1** and detailed spot information is given in **Supplementary Table S2**. In two cases (spots 516 and 1751) more than one protein was found in the excised spots. Since quantification of non-separated proteins is not reliable, these spots were not further investigated. The theoretical isoelectric point and molecular weight of identified proteins matched to the respective spot position on 2D gels to a great extent indicating that full-length polypeptides were identified by the comparative proteome analysis.

**Table 1:** Differentially abundant proteins in *Arabidopsis thaliana* cv. Oy-0 roots identified by mass spectrometry.

Spot number	ANOVA (p)	Fold change	Normalized volumes		Protein accession	Protein description	Protein p*I* (theor./exp.)	Protein MW (theor./exp.)
			Control	Inoculated				
516	0.038	–1.4	8.40E+06	6.07E+06	gi| 15233349	Aconitate hydratase 1 [*A. thaliana*]	6.0/6.2	98.1/105
					gi| 6056373	Elongation factor EF-2 [*A. thaliana*]	5.9/6.2	94.2/105
555	0.042	+1.9	5.17E+05	9.87E(+05	gi| 1495251	Heat shock 70 kDa protein 14 [*A. thaliana*]	5.2/5.2	91.7/100
693	0.009	–1.5	1.14E(+07	7.63E(+06	gi| 15242459	Heat shock 70 kDa protein 10, mitochondrial [*A. thaliana*]	5.6/5.5	72.9/83
972	0.041	–1.3	6.96E(+06	5.31E(+06	gi| 15226573	Ferredoxin-nitrite reductase [*A. thaliana*]	6.0/5.9	65.5/71
1491	0.019	–1.4	2.05E(+06	1.50E(+06	gi| 15231715	Fructose-bisphosphate aldolase, class I [*A. thaliana*]	6.1/5.8	38.5/40
1619	0.037	–1.2	1.89E(+07	1.59E(+07	gi| 30687350	Elongation factor 1-beta 2 [*A. thaliana*]	4.4/4.2	24.2/32
1670	0.009	(+1.4	1.32E(+07	9.45E(+06	gi| 3193303	T14P8.5 [*A. thaliana*]	4.4/4.1	27.9/29
1746	0.02	(+1.4	4.91E(+06	6.98E(+06	gi| 15233268	20S proteasome alpha-3 subunit [*A. thaliana*]	6.6/6.8	27.4/28
1751	0.04	–1.4	2.95E(+06	2.17E(+06	gi| 21553809	Unknown [*A. thaliana*]	4.4/4.0	19.1/26
					gi| 15230476	Nascent polypeptide-associated complex subunit alpha-like protein 1 [*A. thaliana*]	4.3/4.0	21.9/26
1812	0.033	+1.4	6.82E+06	9.40E+06	gi| 15218639	Glutathione *S*-transferase F7 [*A. thaliana*]	6.1/6.8	23.6/24
2239	0.031	–1.3	5.71E+06	4.51E+06	gi| 15236014	Lipase/lipooxygenase [*A. thaliana*]	5.0/4.0	20.1/18
2247	0.042	+1.7	3.22E+06	5.40E+06	gi| 18401345	Universal stress protein family protein [*A. thaliana*]	6.4/6.8	17.7/18

*The table gives the spot number, statistical significance of differential expression, the fold change between groups of control and inoculated samples, protein abundance on 2D gels, the NCBI Viridiplantae database hit and, theoretical and experimental isoelectric points (p*I*) and molecular weight (MW).*

The function of six of the remaining spots was related to stress responses. Increased expression in roots colonized with *K. radicincitans* was found for spots 555 (Heat shock 70 kDa protein 14), 1670 (T14P8.5, a Heat shock 20 kDa-like chaperone), 1812 (Glutathione *S*-transferase F7) and 2247 (a universal stress protein), while decreased expression was observed for spots 693 (Heat shock 70 kDa protein 10) and 2239 (Lipase/Lipooxygenase). Two differentially expressed spots were involved in protein metabolism, one was identified as Elongation factor 1-beta 2 (spot 1619) and reduced in spot intensity in inoculated roots, and the other one was a 20S proteasome alpha-3 subunit (spot 1746) that accumulated in response to the treatment. The remaining two spots were both reduced in expression. Fructose-bisphosphate aldolase (spot 1491) is involved in glycolysis, while Ferredoxin-nitrite reductase (spot 972) is involved in nitrogen assimilation.

### Endophytic Colonization Interferes with Ubiquitin-Dependent Protein Degradation

**FIGURE 3:**
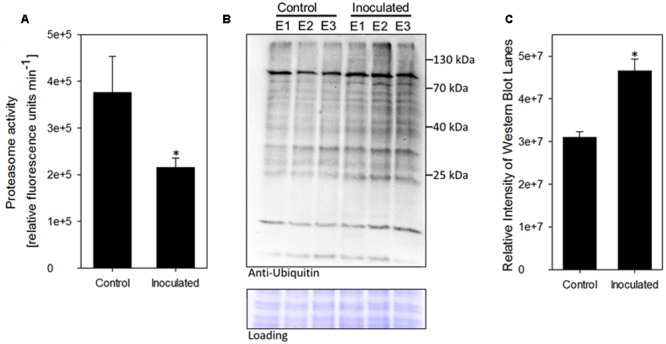
**Presence of the endophyte *K. radicincitans* influences ubiquitin-related protein degradation in *A. thaliana* roots. (A)** Proteasome activity in total protein root extracts was inferred from breakdown of the fluorogenic peptide Suc-LLVY-AMC. Values represent the mean of three independent experiments (±SD). **(B)** Accumulation of ubiquitin conjugates in plant roots upon bacterial colonization. Ubiquitinylated proteins were detected via immunoblotting using an anti-ubiquitin antibody. Equal protein loading was confirmed by Amido-black staining of the western blot membrane. Root protein extracts of three independent experiments (E1–E3) of control and inoculated plants are shown. **(C)** Relative quantification of Western Blot lane intensity using ImageJ. Asterisks indicate statistical significance of differences (*p* < 0.05).

Most of the identified differentially abundant proteins were involved in cellular stress response. Among those, the 20S proteasome alpha-3 subunit (spot 1746) was further investigated. The proteasome is a central hub in protein turn-over, regulating also plant stress and immune responses; however, no correlation to presence of PGPB was drawn in plants so far. Hence, a subsequent analysis of protein degradation properties was carried out to functionally test the observed induction of spot 1746, identified as 20S proteasome alpha-3 subunit. The 20S proteasome represents the core particle of the 26S proteasome. The free 20S proteasome degrades mainly oxidized proteins and RNA, while together with the 19S regulatory particle, the 26S proteasome is formed to degrade proteins in a ubiquitin-dependent manner (Sadanandom et al., 2012). Hence, the accumulation of 20S proteasome alpha-3 subunit could point to an enhanced degradation of oxidized proteins. A second possible explanation for increased levels of 20S proteasome alpha-3 subunit could be a general inhibition of 26S-based protein degradation since it has been shown that the blocking of 26S-related protein degradation results in the accumulation of proteasome subunits (Book et al., 2010; Kim et al., 2013). To test the latter hypothesis, total proteasome activity was determined using a fluorogenic substrate (Suc-LLVY-AMC). Presence of *K. radicincitans* in *A. thaliana* roots led to a 40% inhibition of root proteasome activity as compared to control plants (**Figure 3A**). In order to evaluate the effect of decreased proteasome activity on ubiquitin-mediated protein turnover, immunoblotting analysis using an anti-ubiquitin antibody was performed on total protein extracts of control or inoculated roots. An accumulation of ubiquitinated proteins in roots inoculated with *K. radicincitans* was apparent indicating a disturbed degradation of ubiquitinated proteins in those plants (**Figures 3B,C**).

### 26S Proteasome Regulatory Particle RPN12a Mutant Shows a High Responsiveness toward *K. radicincitans*

**FIGURE 4:**
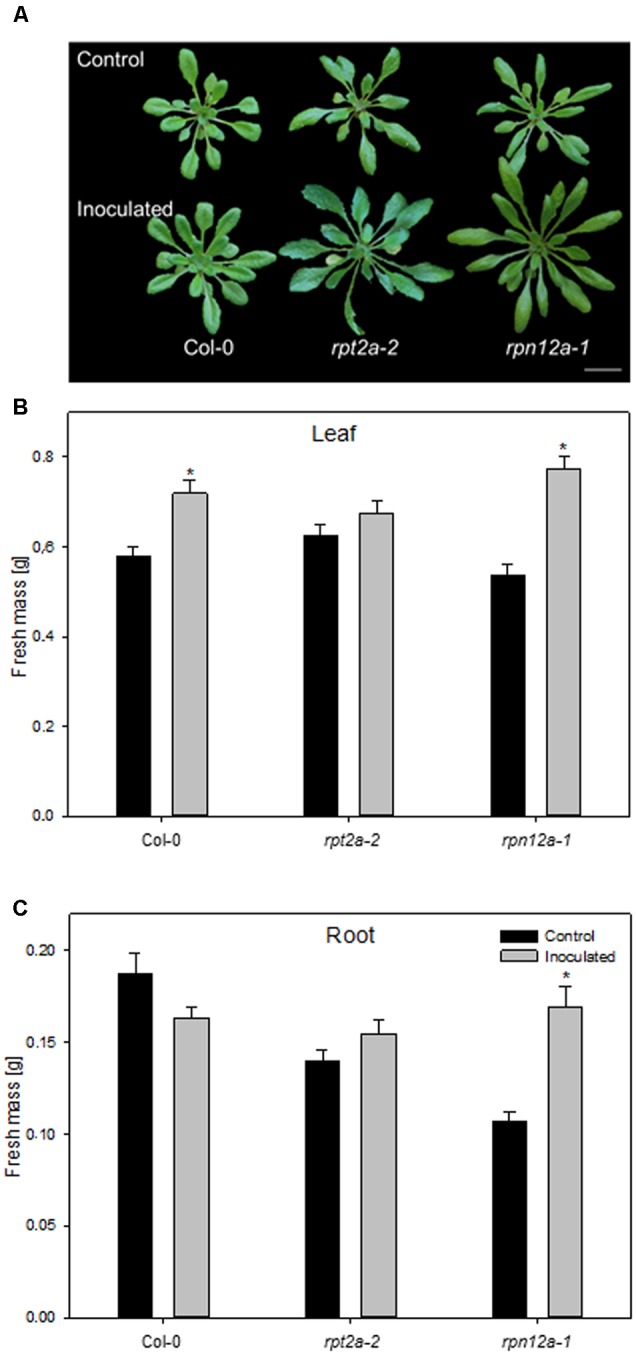
**Impact of *K. radicincitans* presence on phenotype (A)**, and leaf **(B)** and root **(C)** fresh mass of Col-0, *rpt2a-2* and *rpn12a-1*. Values represent the mean ± SE of three independent experiments, each consisting of 25 plants, and the asterisk indicate statistical differences between non-inoculated and inoculated plants (*p* < 0.05). Bar = 2 cm.

The accumulation of ubiquitinated proteins could indicate that the 26S proteasome is affected by the bacterial colonization, rather than the 20S proteasome. In order to unravel the involvement of ubiquitin-dependent or -independent proteolysis in this plant-bacteria interaction, two *A. thaliana* mutants defective in the 26S proteasome regulatory particles RPT2a and RPN12a have been employed to study growth responses upon application of *K. radicincitans*. In those mutants, 26S proteasome activity is strongly reduced, while 20S proteasome levels are increased and the ubiquitin-independent proteolysis is preferentially performed (Kurepa et al., 2008). The effect of bacterial inoculation on plant biomass was tested 4 weeks after inoculation and compared to the Col-0 wild type. The moderate increase in Col-0 biomass production is in agreement with previous observations (Brock et al., 2013). Leaf biomass increased significantly by 24 and 44% in Col-0 and *rpn12a-1*, respectively, compared to non-inoculated plants (**Figure 4**). In roots, a significant effect was observed only for *rpn12a-1* where biomass increased by 58% as compared to the control treatment. This increase is twice as much as found for inoculated Oy-0 roots (see **Figure 1B**). The root and leaf biomass in *rpt2a-2* was increased upon the inoculation. However, those changes were not significant.

## Discussion

The application of PGPB as growth promoters represents a promising strategy in sustainable crop production. However, the modes of physiological alterations provoked by PGPB in plants are less understood. In this work, molecular plant responses to PGPB are investigated to understand adaptation processes resulting from bacterial colonization of the model plant *A. thaliana*. The comparative proteome analysis of plant roots revealed a relatively low number of differentially abundant protein spots between control and inoculated roots with regard to the strong increase in biomass provoked by the bacterium. Most of the identified proteins are involved in cellular stress response. Heat shock proteins are a diverse and complex family of proteins functioning in plant development and in response to environmental stresses, and managing protein folding, cellular trafficking and proteasome targeting (Huang and Xu, 2008; Waters, 2013). The increased abundance of chaperones in roots colonized by *K. radicincitans* indicates a higher demand for protein folding, repair or degradation (McDonough and Patterson, 2003). In this context of protein degradation, a further protein spot with increased abundance in inoculated roots was identified as 20S proteasome alpha-3 subunit. The plant proteasome has been recognized as major target of pathogenic effector proteins (Üstün and Börnke, 2014; Banfield, 2015), but with regard to growth-promoting endophytic bacteria, there are currently no reports that endophytic colonization affects proteasome activity in some way. The manipulation of the host proteasome is an evolutionary conserved virulence mechanism of microbial pathogens that inject effector proteins into the host cell and interfere with proteasome activity in order to suppress immune responses (Üstün et al., 2013, 2014). The relevance of modulating the host proteasome system for non-pathogenic endophytic microbes was demonstrated recently by identifying Syringolin A, a bacterial proteasome inhibitor, in a *Rhizobium* strain isolated from the endosphere of poplar roots (Dudnik et al., 2014). We demonstrated in our study that the colonization of *A. thaliana* by *K. radicincitans* lead to an accumulation of ubiquitinated proteins and is accompanied by a decline in proteasome activity, indicating that the proteasome might be a target for beneficial bacteria as well. Ubiquitination of protein substrates represent the initial step in its proteasome-mediated degradation, induces protein relocalisation or endocytosis. Ubiquitin is a small protein that is covalently linked to a substrate, thereby coordinating phytohormone-related developmental processes, abiotic stress responses and plant immunity (Sadanandom et al., 2012; Banfield, 2015). The increased abundance of chaperones on 2D gels of inoculated roots is correspondingly associated with the disturbed proteolysis, aiming at maintaining cellular processes under *K. radicincitans* colonization. However, no Syringolin A biosynthesis genes or type III secreted effector proteins are present in the *K. radicincitans* genome (Witzel et al., 2012), indicating the possible incidence of other yet unknown effectors.

Currently, opposing observations are discussed in the literature about the abundance of 20S proteasome levels and the ubiquitin-dependent degradation. Using Arabidopsis mutants deficient in regulatory subunits of the 19S regulatory particle, RPT2a and RPN12a, it has been demonstrated, that the reduction in ubiquitin-dependent proteolysis is accompanied by increased levels of 20S proteasome (Kurepa et al., 2008). On the contrary, increased 20S proteasome subunit levels were accompanied by elevated degradation of ubiquitinated proteins was, as shown for the alpha-2 subunit in rice (Li et al., 2015). In our study, the proteasome perturbations induced by *K. radicincitans* were further characterized using mutants deficient in 19S regulatory particle subunits. RPT2a is a triple ATPase involved in gametogenesis, sugar response, root and shoot apical meristem maintenance, and histone dynamics (Kurepa et al., 2009; Lee et al., 2011; Ueda et al., 2011; Sun et al., 2012). RPN12a has no ATPase activity and acts as a negative regulator of cytokinin signaling (Ryu et al., 2009). Both mutants exhibit a decrease in 26S proteasome activity (app. 30% in *rpt2a* and 50% in *rpn12a-1*) but an increase in 20S proteasome activity (Kurepa et al., 2008). The different response of the two mutant genotypes to inoculation with *K. radicincitans* indicates that inhibition of proteasomal turnover *per se* is not sufficient to increase plant biomass in the presence of the bacterium. This could be due to the different extent of proteasome activity inhibition in the mutants. Our own measurements indicate that proteasome activity upon *K. radicincitans* inoculation decreases by app. 50%, which is similar to what was observed in the *rpn12a-1* mutant (Kurepa et al., 2008). Possibly a lower degree of inhibition as in the *rpt2a-2* mutant is not sufficient to trigger a growth response upon bacterial inoculation. Another explanation could be a functional specialization of the regulatory particle subunits under study. The massive biomass accumulation in *rpt12a* upon endophyte colonization, which was even higher than in Oy-0, could be indicative that a strong reduction of 26S proteasome activity is beneficial for *K. radicincitans*-induced plant growth promotion. Although both mutants display a general inhibition of proteasomal protein turnover, they display different phenotypes. Thus, it has been suggested that some subunits recognize a distinct subset of targets, and are therefore responsible for the regulation of a specific developmental or hormonal pathway (Smalle et al., 2002, 2003; Ueda et al., 2004). Hence, another explanation for the different response to colonization of the two mutant phenotypes could be that a defect in RPN12a specifically inhibits processes or pathways that have a negative effect on bacterial colonization or supports bacterial growth promotion by enhancing specific cellular processes which are required for the growth promoting effect of *K. radicincitans*. Whether the bacterial-induced manipulation of the host proteasome functioning is causal for the growth promotion effect or if it is a prerequisite for successful plant colonization needs to be verified in future studies. While for plant pathogens numerous effector proteins as well as their host target are described, information for beneficial endophytes is scarce. The genome of *K. radicincitans* comprises of several genes coding for components of types I, II, IV, and VI secretion system and it is likely that bacterial proteins are targeted to the plant cell cytosol. Generation of functional knock-outs of putative effector proteins will provide more information on the molecular basis of perturbations in the host ubiquitin system.

## Conclusion

By comparing the root proteome of inoculated and non-inoculated plants, we have been able to identify the plant proteasome as essential for establishing a beneficial interaction between *K. radicincitans* and *A. thaliana*. Influencing cellular protein degradation is an efficient virulence strategy of plant pathogens, but it is yet unclear whether this also applies to beneficial plant-bacteria interactions or if proteasome activity is influenced by other factors. Future studies will focus on identifying the mechanistic basis of how proteasome function is altered by *K. radicincitans*, e.g., by identifying effector proteins, and whether this is necessary to overcome plant defense during a successful colonization or whether it can be related to the growth promoting effect of the bacterium.

## Author Contributions

The experiments were conceived and designed by KW, SÜ, MS, RG, FB, and SR. KW and SÜ performed the experiments and analyzed the data. KW, SÜ, MS, RG, FB, and SR wrote the paper.

## Conflict of Interest Statement

The authors declare that the research was conducted in the absence of any commercial or financial relationships that could be construed as a potential conflict of interest.

## Footnotes

1 https://imagej.nih.gov/ij/index.html

## Supplementary Material

TABLE S1 **The identification of protein spots.** For MS/MS-based identifications, the Mascot protein score, observed peptide masses, peptide tolerance in ppm, missed cleavages, the Mascot peptide score and the respective *de novo* peptide sequences are given.

TABLE S2 **Summary of 2D experiments.** Presented are the reference gel image and outlines of the spots with annotation, a table containing a summary of all statistical significantly regulated spots (spot number, result of ANOVA analysis, maximum fold change, and averaged normalized spot volumes), and details of each spot, including expression profile and image montage.
