# Supplementary material for: A Proteomic Approach Suggests Unbalanced Proteasome Functioning Induced by the Growth-Promoting Bacterium Kosakonia radicincitans in Arabidopsis
Source: Front Plant Sci. 2017 Apr 26;8:661. doi: 10.3389/fpls.2017.00661 (PMC5405128; doi:10.3389/fpls.2017.00661)

**Reference image**


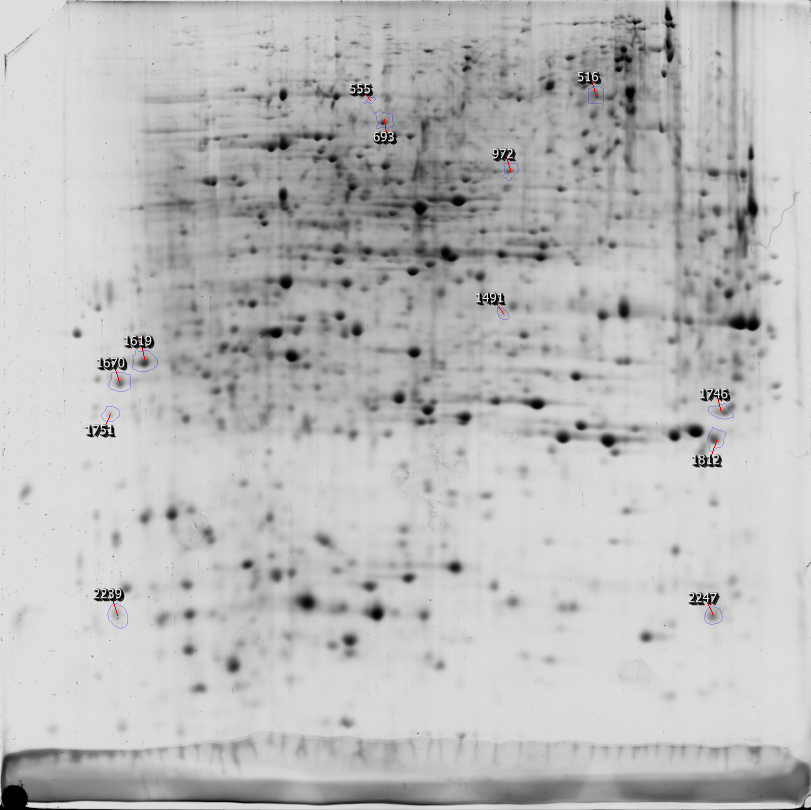


**Spots**

| **#** | **Anova (p)** | **Fold** | **Average Normalised Volumes** | |
| --- | --- | --- | --- | --- |
|  |  |  | **control** | **inoculated** |
| 693 | 0,009 | 1,5 | 1,141e+007 | 7,634e+006 |
| 1670 | 0,009 | 1,4 | 1,321e+007 | 9,449e+006 |
| 1491 | 0,019 | 1,4 | 2,053e+006 | 1,498e+006 |
| 1746 | 0,020 | 1,4 | 4,914e+006 | 6,979e+006 |
| 2239 | 0,031 | 1,3 | 5,713e+006 | 4,507e+006 |
| 1812 | 0,033 | 1,4 | 6,823e+006 | 9,397e+006 |
| 1619 | 0,037 | 1,2 | 1,890e+007 | 1,585e+007 |
| 516 | 0,038 | 1,4 | 8,401e+006 | 6,072e+006 |
| 1751 | 0,040 | 1,4 | 2,950e+006 | 2,174e+006 |
| 972 | 0,041 | 1,3 | 6,957e+006 | 5,314e+006 |
| 555 | 0,042 | 1,9 | 5,168e+005 | 9,874e+005 |
| 2247 | 0,042 | 1,7 | 3,221e+006 | 5,404e+006 |

**Identifier 693**

| **Position** | (975, 311) |
| --- | --- |
| **Notes** |  |

| **control** | **inoculated** |
| --- | --- |
| 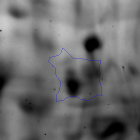 | 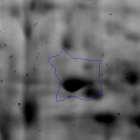 |


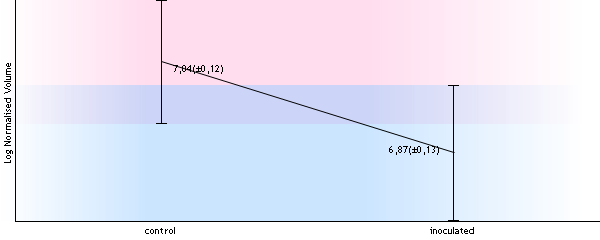


**Identifier 1670**

| **Position** | (305, 975) |
| --- | --- |
| **Notes** |  |

| **control** | **inoculated** |
| --- | --- |
| 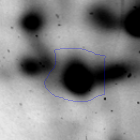 | 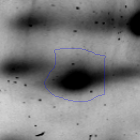 |


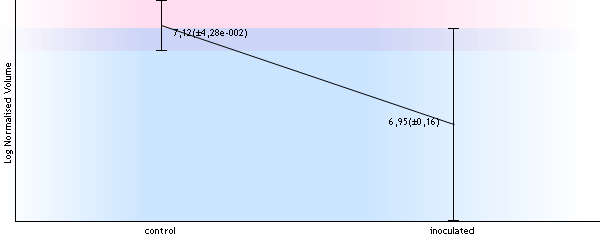


**Identifier 1491**

| **Position** | (1282, 791) |
| --- | --- |
| **Notes** |  |

| **control** | **inoculated** |
| --- | --- |
| 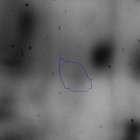 | 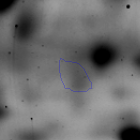 |


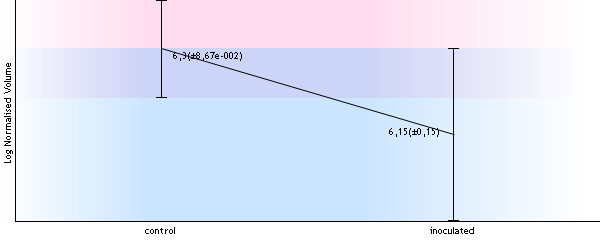


**Identifier 1746**

| **Position** | (1846, 1048) |
| --- | --- |
| **Notes** |  |

| **control** | **inoculated** |
| --- | --- |
| 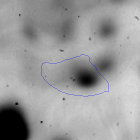 | 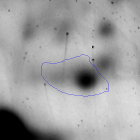 |


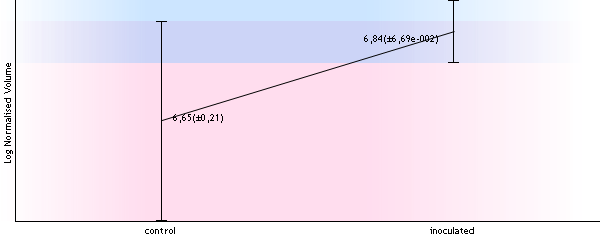


**Identifier 2239**

| **Position** | (297, 1561) |
| --- | --- |
| **Notes** |  |

| **control** | **inoculated** |
| --- | --- |
| 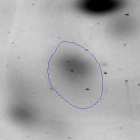 | 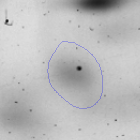 |


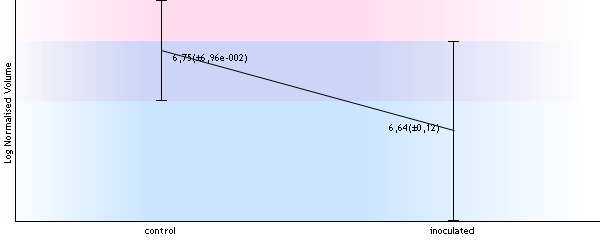


**Identifier 1812**

| **Position** | (1817, 1118) |
| --- | --- |
| **Notes** |  |

| **control** | **inoculated** |
| --- | --- |
| 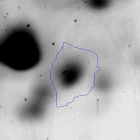 | 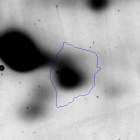 |


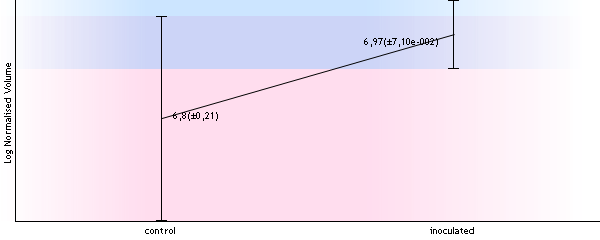


**Identifier 1619**

| **Position** | (367, 924) |
| --- | --- |
| **Notes** |  |

| **control** | **inoculated** |
| --- | --- |
| 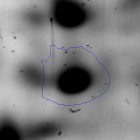 | 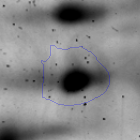 |


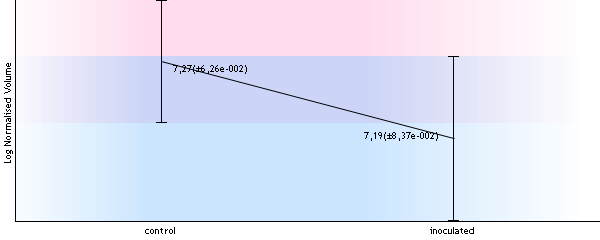


**Identifier 516**

| **Position** | (1518, 244) |
| --- | --- |
| **Notes** |  |

| **control** | **inoculated** |
| --- | --- |
| 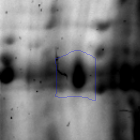 | 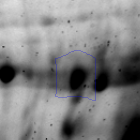 |


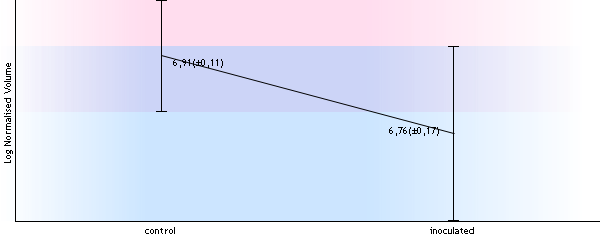


**Identifier 1751**

| **Position** | (278, 1055) |
| --- | --- |
| **Notes** |  |

| **control** | **inoculated** |
| --- | --- |
| 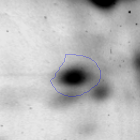 | 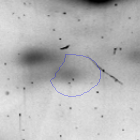 |


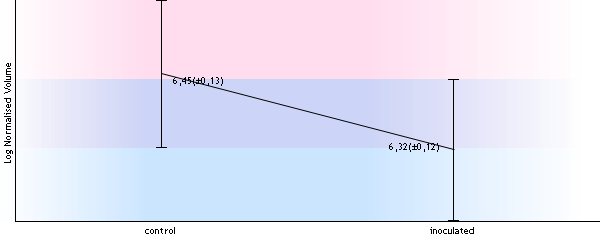


**Identifier 972**

| **Position** | (1293, 436) |
| --- | --- |
| **Notes** |  |

| **control** | **inoculated** |
| --- | --- |
| 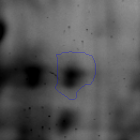 | 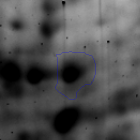 |


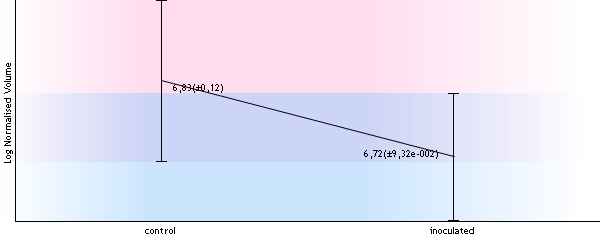


**Identifier 555**

| **Position** | (940, 255) |
| --- | --- |
| **Notes** |  |

| **control** | **inoculated** |
| --- | --- |
| 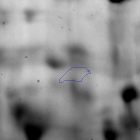 | 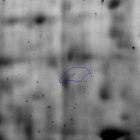 |


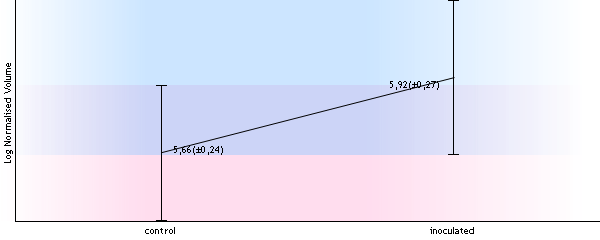


**Identifier 2247**

| **Position** | (1814, 1567) |
| --- | --- |
| **Notes** |  |

| **control** | **inoculated** |
| --- | --- |
| 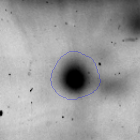 | 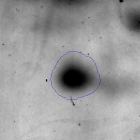 |


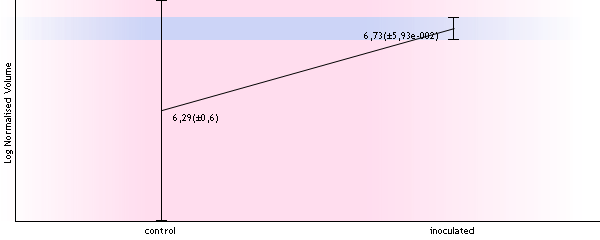

Supplement: TABLE S2 — Summary of 2D experiments. Presented are the reference gel image and outlines of the spots with annotation, a table containing a summary of all statistical significantly regulated spots (spot number, result of ANOVA analysis, maximum fold change, and averaged normalized spot volumes), and details of each spot, including expression profile and image montage. [file Data_Sheet_2.DOCX]
